# Supplementary material for: Generation and characterization of monoclonal antibodies against pathologically phosphorylated TDP-43
Source: PLoS One. 2024 Apr 18;19(4):e0298080. doi: 10.1371/journal.pone.0298080 (PMC11025846; doi:10.1371/journal.pone.0298080)
Supplement: S1 Fig — (DOCX) [file pone.0298080.s001.docx]

**
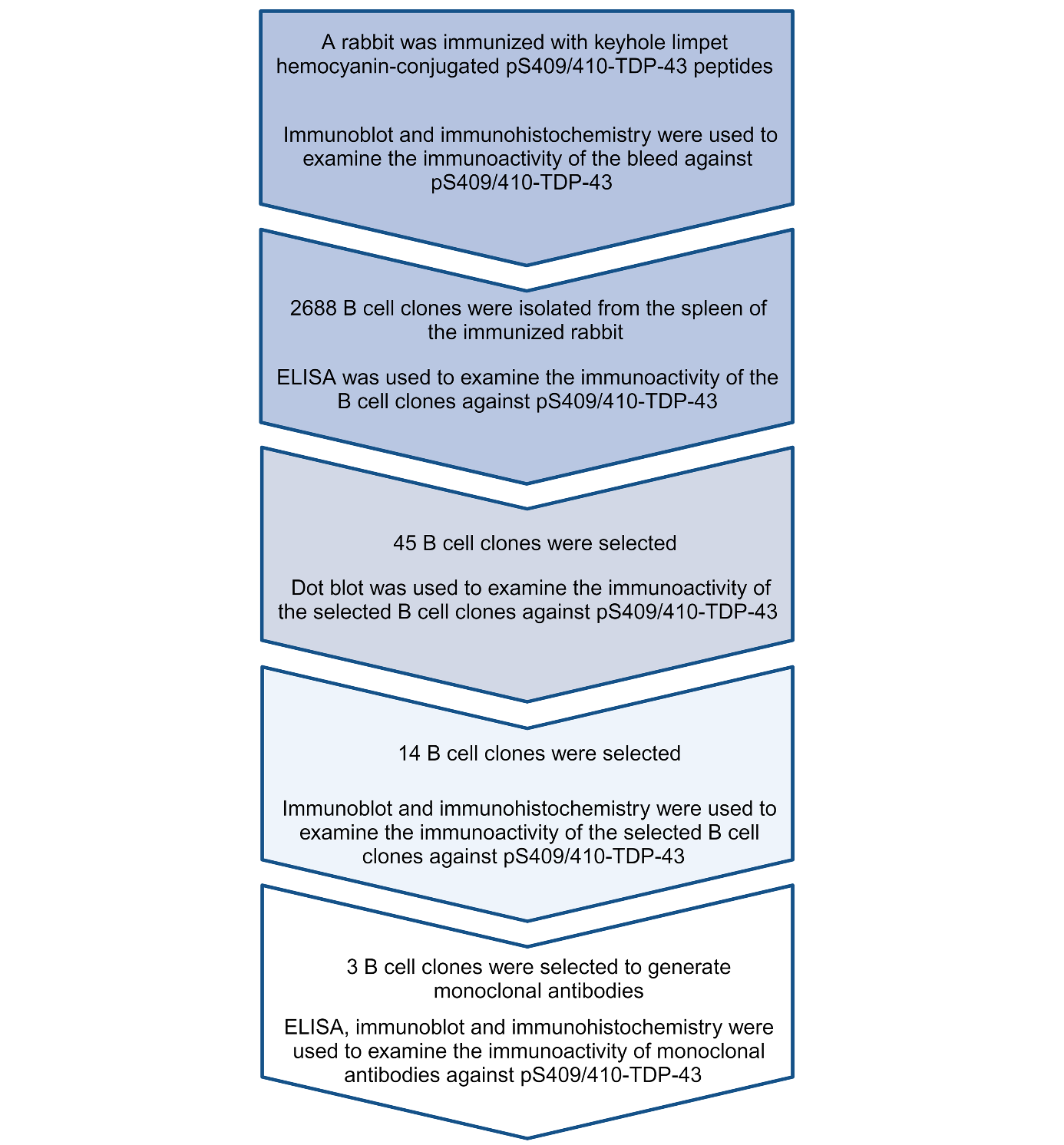
**

**Fig S1. Schematic of the methods and procedures to generate, screen and characterize monoclonal antibodies against pS409/410-TDP-43.** Schematic created with BioRender.com.
