# Supplementary material for: Generation and characterization of monoclonal antibodies against pathologically phosphorylated TDP-43
Source: PLoS One. 2024 Apr 18;19(4):e0298080. doi: 10.1371/journal.pone.0298080 (PMC11025846; doi:10.1371/journal.pone.0298080)
Supplement: S2 Fig — (DOCX) [file pone.0298080.s002.docx]

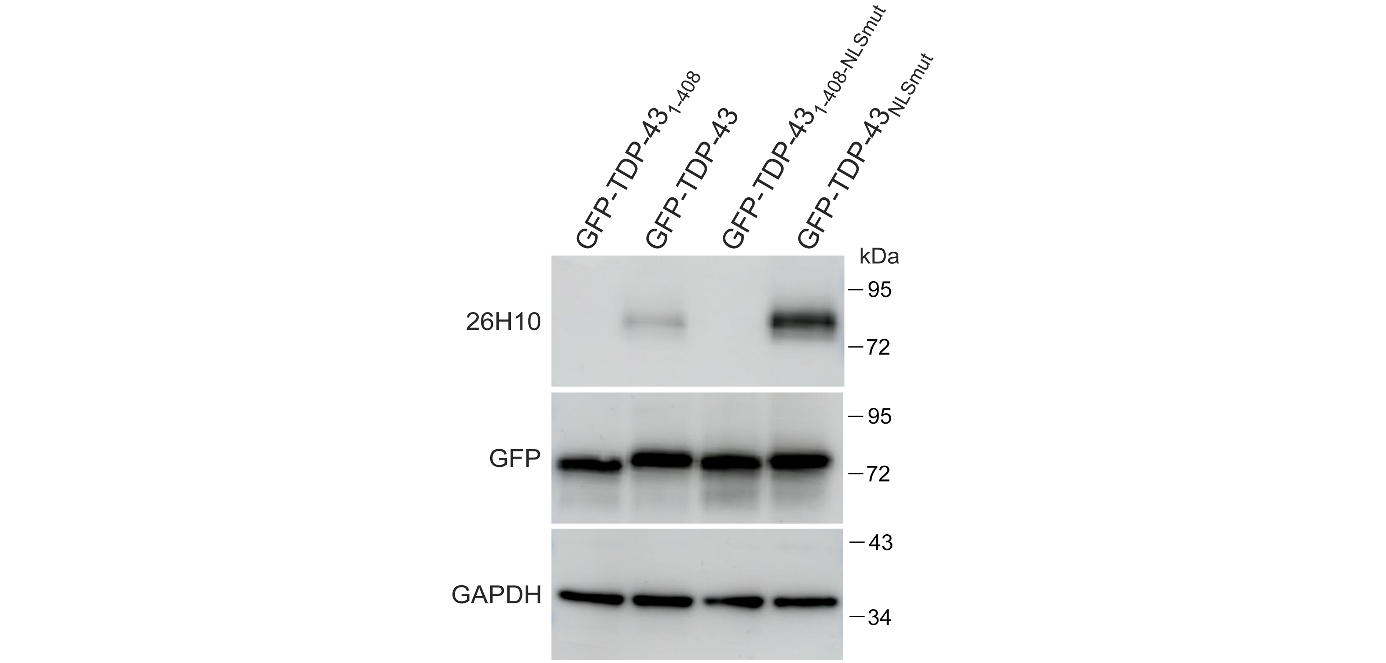


**Fig S2. 26H10 rabbit mAb exhibits high specificity to pS409/410-TDP-43.** Immunoblot analysis of HEK293T cell lysates expressing GFP-TDP-43_1-408_, GFP-TDP-43, GFP-TDP-43_1-408-NLSmut_, or GFP-TDP-43_NLSmut_ using the indicated antibodies. GAPDH was used as a loading control.
