# Supplementary material for: Generation and characterization of monoclonal antibodies against pathologically phosphorylated TDP-43
Source: PLoS One. 2024 Apr 18;19(4):e0298080. doi: 10.1371/journal.pone.0298080 (PMC11025846; doi:10.1371/journal.pone.0298080)
Supplement: S3 Fig — (DOCX) [file pone.0298080.s003.docx]

**
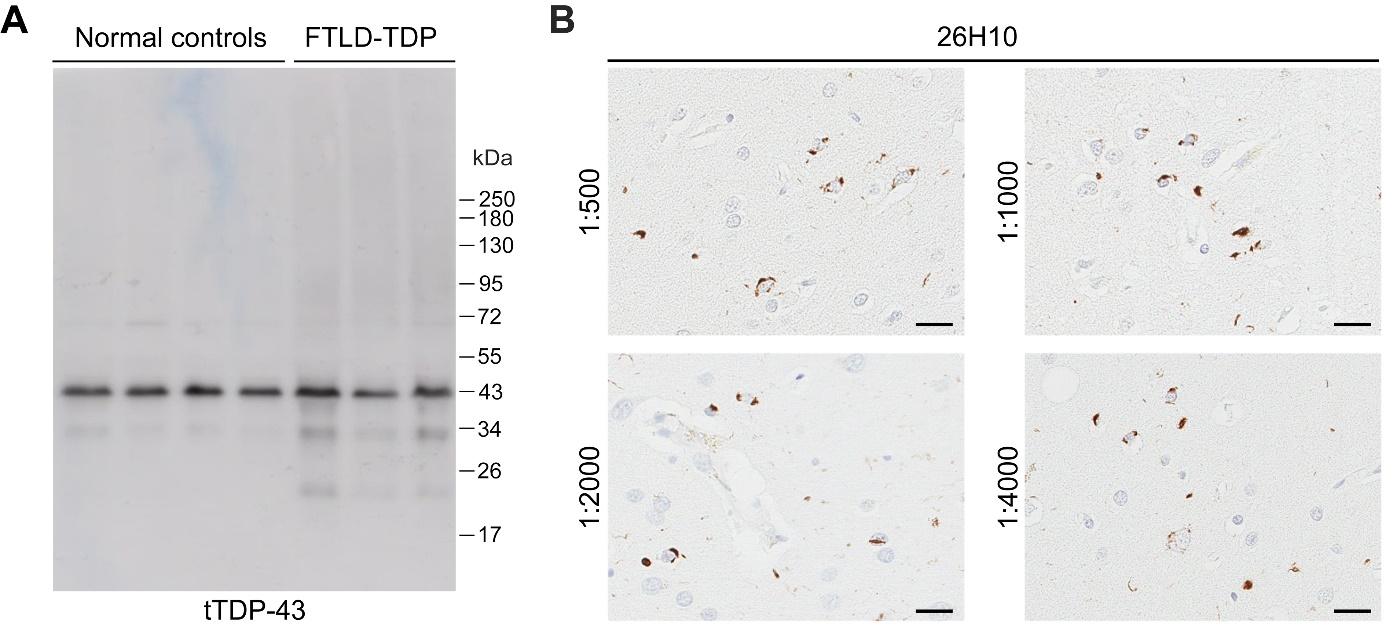
**

**Fig S3. 26H10 rabbit mAb detects TDP-43 pathology in FTLD-TDP brain tissues.** (A) Immunoblot analysis of urea-soluble fractions from the frontal cortex of FTLD-TDP patients and normal controls using an antibody against total TDP-43 (tTDP-43). (B) Representative images of immunohistochemical analysis using different dilutions of the 26H10 rabbit mAb in the frontal cortex of FTLD-TDP patients. Scale bars are 20 µm.
