# Supplementary material for: Generation and characterization of monoclonal antibodies against pathologically phosphorylated TDP-43
Source: PLoS One. 2024 Apr 18;19(4):e0298080. doi: 10.1371/journal.pone.0298080 (PMC11025846; doi:10.1371/journal.pone.0298080)
Supplement: S1 Table — (DOCX) [file pone.0298080.s004.docx]

**Table S1. Characteristics of patients with FTD/ALS**

| **Case #** | **Pathological Diagnosis** | **Gender** | **Age at Onset** | **Age at death** | **Disease**  **Duration** |
| --- | --- | --- | --- | --- | --- |
| 1 | Normal | M | n.a. | 82 | n.a. |
| 2 | Normal | M | n.a. | 80 | n.a. |
| 3 | Normal | F | n.a. | 65 | n.a. |
| 4 | Normal | M | n.a. | 79 | n.a. |
| 5 | Normal | M | n.a. | 88 | n.a. |
| 6 | FTLD-TDP | F | 75 | 83 | 8 |
| 7 | FTLD-TDP | M | 55 | 66 | 11 |
| 8 | FTLD-TDP | M | 76 | 85 | 9 |
| 9 | FTLD-TDP/ALS | F | 64 | 66 | 2 |
| 10 | ALS | M | 63 | 64 | 1 |
| 11 | FTLD-TDP | M | 54 | 60 | 6 |
| 12 | FTLD-TDP | F | 76 | 85 | 9 |
| 13 | FTLD-TDP | M | 78 | 87 | 9 |
| 14 | FTLD-TDP | F | 84 | 86 | 2 |

FTD, frontotemporal degeneration; ALS, amyotrophic lateral sclerosis
