# Supplementary material for: Generation and characterization of monoclonal antibodies against pathologically phosphorylated TDP-43
Source: PLoS One. 2024 Apr 18;19(4):e0298080. doi: 10.1371/journal.pone.0298080 (PMC11025846; doi:10.1371/journal.pone.0298080)
Supplement: S2 Table — (DOCX) [file pone.0298080.s005.docx]

| **Table S2. Immunoreactivity of B cell clones against TDP-43 species as measured by ELISA immunoassay** | | | | |
| --- | --- | --- | --- | --- |
|  | **Primary screening** | **Counter screening** | | |
| **Clone #** | **TDP-43 pS409/410-Biotin** | **TDP-43 pS409-Biotin** | **TDP-43 pS410-Biotin** | **TDP-43 NP-Biotin** |
| 23A1 | 2.382 | 0.828 | 0.103 | 0.082 |
| 22B6 | 2.198 | 1.203 | 0.221 | 0.067 |
| 21B6 | 2.173 | 1.053 | 0.862 | 0.242 |
| 22E2 | 2.109 | 0.936 | 0.098 | 0.072 |
| 22A10 | 1.969 | 0.742 | 0.688 | 0.168 |
| 22C1 | 1.933 | 0.989 | 0.726 | 0.158 |
| 26A3 | 1.922 | 0.92 | 1.123 | 0.631 |
| 24B8 | 1.877 | 0.007 | 0.182 | 0.058 |
| 17H4 | 1.758 | 0.153 | 0.047 | 0.029 |
| 24F2 | 1.725 | 0.714 | 0.269 | 0.059 |
| 23G8 | 1.72 | 0.904 | 0.685 | 0.087 |
| 22B7 | 1.718 | 0.472 | 0.884 | 0.194 |
| 25F7 | 1.716 | 0.685 | 0.63 | 0.1 |
| 4A4 | 1.692 | 0.272 | 0.204 | 0.045 |
| 27C1 | 1.647 | 0.745 | 0.754 | 0.119 |
| 27C6 | 1.642 | 0.869 | 0.644 | 0.135 |
| 26B7 | 1.628 | 1.133 | 1.315 | 0.914 |
| 24H5 | 1.618 | 1.079 | 1.174 | 0.593 |
| 24C8 | 1.611 | 0.006 | 0.232 | 0.143 |
| 27A4 | 1.6 | 0.601 | 0.83 | 0.179 |
| 21G4 | 1.597 | 0.666 | 0.461 | 0.06 |
| 2E9 | 1.55 | 0.123 | 0.053 | 0.024 |
| 24H8 | 1.526 | 0.825 | 0.809 | 0.121 |
| 27D5 | 1.526 | 0.499 | 0.161 | 0.025 |
| 21C12 | 1.517 | 0.705 | 0.632 | 0.115 |
| 26B4 | 1.509 | 0.405 | 0.527 | 0.306 |
| 4F3 | 1.465 | 0.215 | 0.209 | 0.047 |
| 21D9 | 1.454 | 0.606 | 0.708 | 0.081 |
| 26H10 | 1.435 | 0.802 | 0.168 | 0.112 |
| 25E7 | 1.432 | 0.727 | 1.064 | 0.352 |
| 23C1 | 1.39 | 0.376 | 0.034 | 0.055 |
| 27E11 | 1.386 | 0.962 | 1.149 | 0.809 |
| 21E2 | 1.357 | 0.284 | 0.056 | 0.014 |
| 21E10 | 1.338 | 0.337 | 0.545 | 0.104 |
| 17A4 | 1.334 | 0.252 | 0.186 | 0.035 |
| 2G6 | 1.23 | 0.135 | 0.057 | 0.209 |
| 23A8 | 1.226 | 0.374 | 0.031 | 0.021 |
| 4F7 | 1.155 | 0.143 | 0.108 | 0.073 |
| 2H2 | 1.146 | 0.156 | 0.055 | 0.029 |
| 24A5 | 1.146 | 0.307 | 0.289 | 0.038 |
| 4B7 | 1.13 | 0.187 | 0.066 | 0.086 |
| 1B5 | 1.113 | 0.033 | 0.043 | 0.019 |
| 23C6 | 1.096 | 0.611 | 0.484 | 0.072 |
| 23F2 | 1.039 | 0.189 | 0.011 | 0.014 |
| 23H11 | 1.022 | 0.704 | 0.246 | 0.093 |
| 7A8 | 0.99 | 0.124 | 0.194 | 0.126 |
| 24D10 | 0.96 | 0.151 | 0.024 | 0.028 |
| 5F10 | 0.953 | 0.23 | 0.376 | 0.145 |
| 23A5 | 0.896 | 0.363 | 0.304 | 0.049 |
| 24D6 | 0.882 | 0.005 | 0.038 | 0.063 |
| 27B4 | 0.866 | 0.037 | 0.014 | 0.008 |
| 21F3 | 0.85 | 0.304 | 0.04 | 0.009 |
| 23B6 | 0.755 | 0.207 | 0.028 | 0.016 |
| 2B3 | 0.727 | 0.178 | 0.256 | 0.129 |
| 22F8 | 0.714 | 0.12 | 0.017 | 0.043 |
| 23C7 | 0.651 | 0.1 | 0.043 | 0.011 |
| 23B12 | 0.58 | 0.06 | 0.013 | 0.008 |
| 23G3 | 0.57 | 0.016 | 0.061 | 0.011 |
| 9G11 | 0.559 | 0.124 | 0.049 | 0.04 |
| 2H6 | 0.551 | 0.122 | 0.042 | 0.039 |
| 21A9 | 0.533 | 0.058 | 0.009 | 0.005 |
| 5G7 | 0.519 | 0.019 | 0.007 | 0.022 |
| 23E8 | 0.502 | 0.093 | 0.014 | 0.01 |
| 22H3 | 0.483 | 0.094 | 0.014 | 0.003 |
| 21C3 | 0.481 | 0.076 | 0.019 | 0.008 |
| 4D1 | 0.48 | 0.024 | 0.006 | 0.078 |
| 24G8 | 0.477 | 0.375 | 0.12 | 0.036 |
| 22B2 | 0.475 | 0.026 | 0.02 | 0.007 |
| 23E1 | 0.468 | 0.102 | 0.01 | 0.032 |
| 24E6 | 0.466 | 0.089 | 0.013 | 0.007 |
| 23H9 | 0.464 | 0.063 | 0.018 | 0.007 |
| 23B10 | 0.452 | 0.114 | 0.014 | 0.012 |
| 1F8 | 0.436 | 0.028 | 0.013 | 0.03 |
| 2H4 | 0.427 | 0.008 | 0.012 | 0.007 |
| 24D8 | 0.42 | 0.028 | 0.015 | 0.008 |
| 21A1 | 0.419 | 0.079 | 0.011 | 0.008 |
| 2C9 | 0.391 | 0.091 | 0.056 | 0.035 |
| 1C10 | 0.381 | 0.155 | 0.056 | 0.037 |
| 23E11 | 0.379 | 0.039 | 0.007 | 0.021 |
| 9H7 | 0.378 | 0.007 | 0.006 | 0.013 |
| 23B2 | 0.378 | 0.039 | 0.016 | 0.005 |
| 2H7 | 0.37 | 0.036 | 0.011 | 0.008 |
| 9H2 | 0.366 | 0.058 | 0.008 | 0.009 |
| 23H1 | 0.358 | 0.076 | 0.012 | 0.006 |
| 2D4 | 0.348 | 0.037 | 0.018 | 0.021 |
| 19E2 | 0.327 | 0.103 | 0.006 | 0.007 |
| 24H12 | 0.321 | 0.03 | 0.011 | 0.011 |
| 2E12 | 0.312 | 0.029 | 0.006 | 0.013 |
| 5B2 | 0.308 | 0.02 | 0.012 | 0.009 |
