# Supplementary figures and images for: Generation and characterization of monoclonal antibodies against pathologically phosphorylated TDP-43
Source: PLoS One. 2024 Apr 18;19(4):e0298080. doi: 10.1371/journal.pone.0298080 (PMC11025846; doi:10.1371/journal.pone.0298080)

Figure 1A

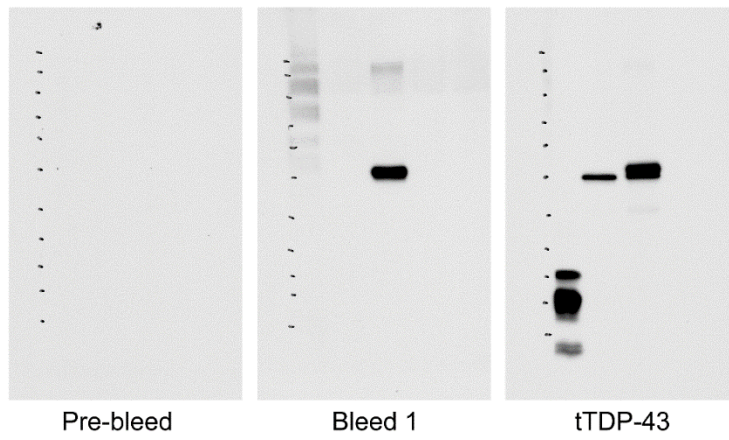

Figure 1B

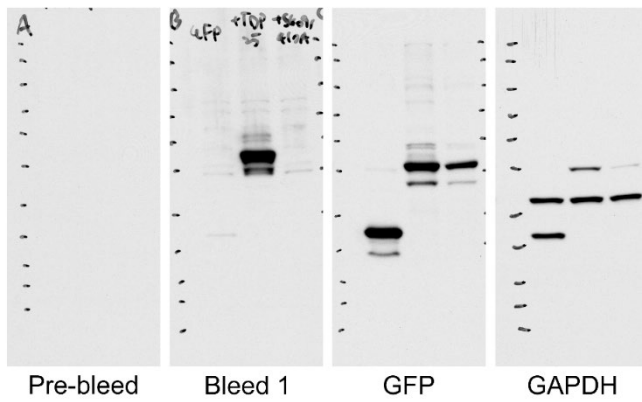

Figure 2A

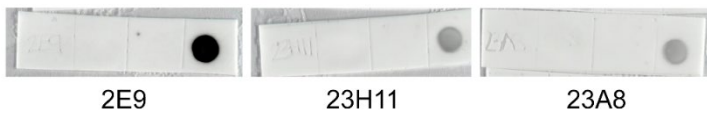

Figure 2B

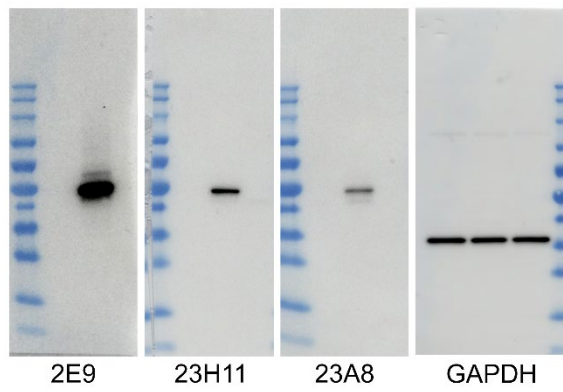

Figure 3B

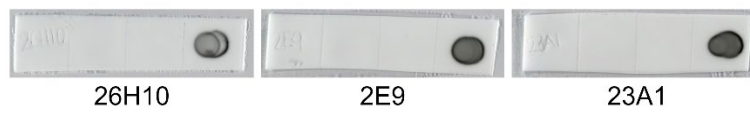

Figure 3C

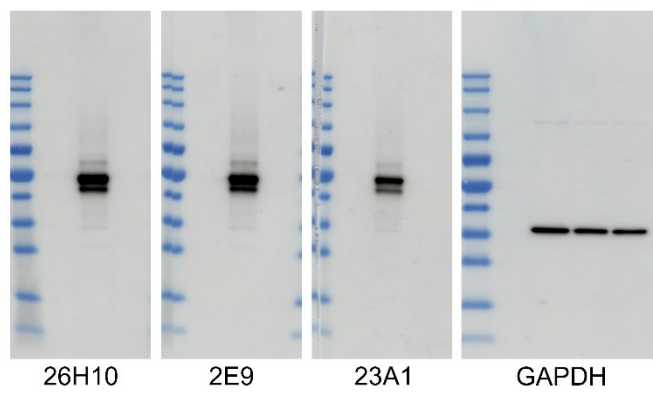

Figure 4A

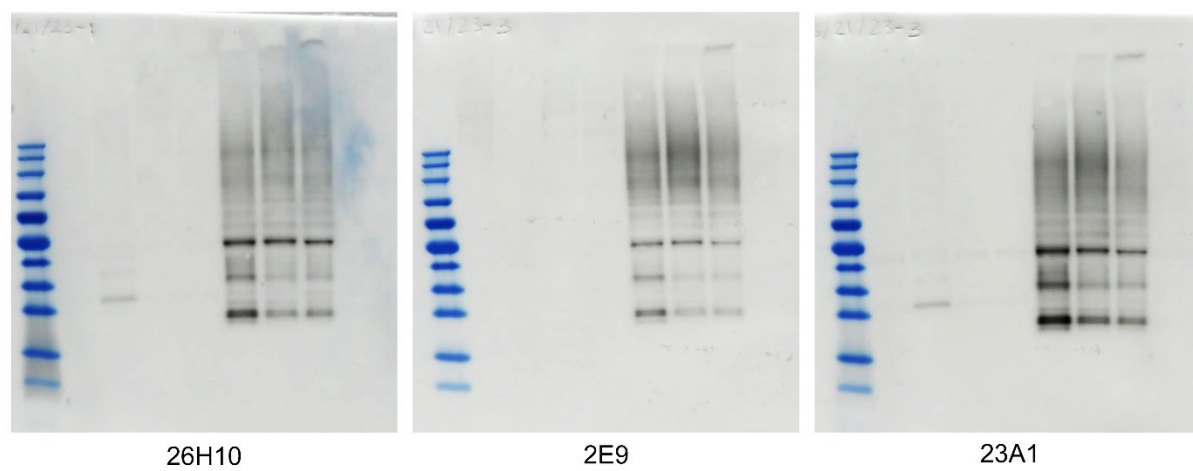

Figure S2

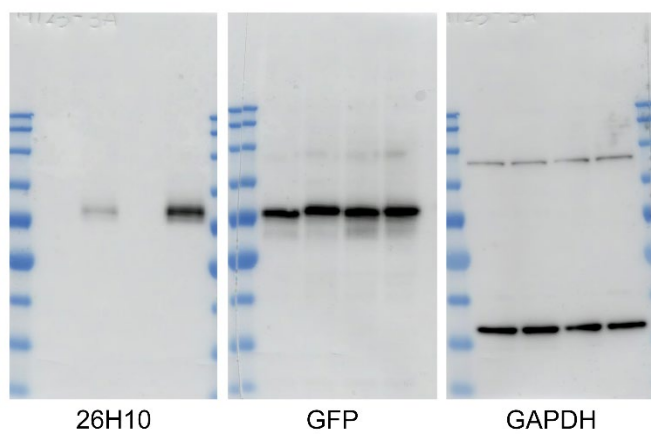

Figure S3A

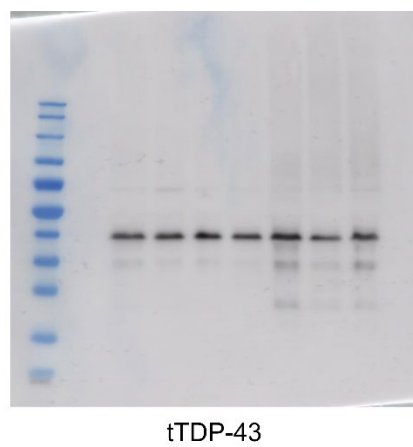

Supplement: S1 Raw images — (PDF) [file pone.0298080.s006.pdf]
