# Supplementary material for: Generation and characterization of monoclonal antibodies against pathologically phosphorylated TDP-43
Source: PLoS One. 2024 Apr 18;19(4):e0298080. doi: 10.1371/journal.pone.0298080 (PMC11025846; doi:10.1371/journal.pone.0298080)
Supplement: Attachment — Submitted filename: Zhang et al review.pdf [file pone.0298080.s007.pdf]

In this brief manuscript, Zhang et al. describe the generation of novel antibodies directed against phospho-TDP-43 and provide compelling evidence of the antibodies´ validity detecting TDP-43 inclusions in situ. The data is clearly presented and the conclusions appear robust. The material presented here could be of interest and use in the field and the manuscript should be of interest to the reader. However, the manuscript could be further elaborated to provide a clear discussion positioning the novel antibodies with respect to the available ones and additional experiments could help to detail which species are really being detected. What follows is a list of issues which, in this reviewer´s opinion, could help improve the manuscript and categorize the described antibodies.

Major points:

#1. Please provide a clear description of the advantages of these novel antibodies with respect to the commercial ones, in particular in light of recent publications (Riemenschneider et al. https://doi.org/10.1186/s40478-023-01592-z).

#2. The major confusion raised while following the manuscript was trying to understand which species are specifically targeted by the antibodies. Although still open to debate, it could be concluded that phosphorylation of TDP-43 low complexity domain promotes insolubility (see da Silva et al. doi: 10.15252/embj.2021108443). In addition, CK1 phosphorylation may not be complete, which means that the antigens for immunization could correspond to aggregates containing mixtures of phosphorylated and non-phosphorylated TDP-43. In Figure 2, for instance, it is not clear if cell B supernatant is targeting aggregated (urea-soluble, in a broad way) rather than selectively phosphorylated TDP-43. In addition, Figure 4 suggests that the novel antibodies are targeting aggregated TDP-43. To clarify this, authors should demonstrate that aggregated, non-phosphorylated TDP-43 is not detected by the novel antibodies.

#3. Authors generate a double Ala mutant in positions 409/410 as a control of phosphorylation of these specific sites. Instead, authors should test the possible detection by the antibodies of a truncated version of TDP-43, where both 409 and 410 (and beyond) sites are not included.

Minor points:
#1. Please provide an explanatory scheme of the procedure followed to produce the antibodies and the specific epitopes which are targeted.

#2. Please include the sequence of the biotinylated peptides used in the dot-blots in Fig.2.

#3. Please include a loading control in the western blots (Calnexin, b-actin).

#4. Antibodies are used in 1:500 dilution in immunochemistry experiments. This appears too high. Please provide an explanation.

#5. Please show the uncropped gel in Fig. 1A, or at least the region covering molecular weights below 34 kDa. This is particularly relevant in Fig. 1B since the data with the doble Ala mutant appears crucial.

#6. In lines 219-220, reference to Fig 1B should instead be Fig 1C.
