# Supplementary material for: Generation and characterization of monoclonal antibodies against pathologically phosphorylated TDP-43
Source: PLoS One. 2024 Apr 18;19(4):e0298080. doi: 10.1371/journal.pone.0298080 (PMC11025846; doi:10.1371/journal.pone.0298080)
Supplement: Attachment — Submitted filename: Response to Reviewers_FINAL.docx [file pone.0298080.s008.docx]

We appreciate all the insightful comments, positive feedback and enthusiasm provided by the reviewer and editor. Please find below a point-by-point description of how we have addressed each concern.

**Reviewer's comments:**

Reviewer: In this brief manuscript, Zhang et al. describe the generation of novel antibodies directed against phospho-TDP-43 and provide compelling evidence of the antibodies’ validity detecting TDP-43 inclusions in situ. The data is clearly presented and the conclusions appear robust. The material presented here could be of interest and use in the field and the manuscript should be of interest to the reader.

*Response: We appreciate the reviewer’s enthusiasm for our manuscript.*

Major points:

1. Please provide a clear description of the advantages of these novel antibodies with respect to the commercial ones, in particular in light of recent publications (Riemenschneider et al. https://doi.org/10.1186/s40478-023-01592-z).

*Response:* *We thank the reviewer for the suggestion. Compared to commercial antibodies* *against phosphorylated TDP-43 at serines 409/410 (pS409/410-TDP-43), our antibodies have two major advantages. First, our antibodies have been comprehensively characterized. We showed that our antibodies exhibit high specificity and sensitivity against pS409/410-TDP-43 and are ideal for use in multiple assays including biochemistry, immunohistochemistry, and immunoassays. We also showed that our antibodies can be applied to multiple model systems including recombinant proteins, cultured cells, mouse tissues and human samples.* *Second, our antibodies are rabbit monoclonal antibodies, while commercial rabbit antibodies are polyclonal. We have the plasmids for the heavy and light chain of each antibody and transfected these plasmids into Expi293F cells to produce our purified antibodies. Therefore, our antibodies will show high reproducibility with minimal batch effects. Further, while commercial monoclonal pS409/410-TDP-43 antibodies are also available, they are derived from mice and therefore may not be ideal in some applications. We revised our manuscript to include these two points in the Discussion section.*

*To address the second part of the reviewer’s comment: Riemenschneider et al. recently tested the safety and efficacy of multiple TDP-43 peptides in a TDP-43 mouse model to evaluate their potentials as the passive immunotherapy. They showed that immunization with pS409/410-TDP-43 peptides – specifically CSMDSK[pS][pS]GWGM, the same peptide used in our manuscript – reduces neuroaxonal damage. Thus, the results included in the Riemenschneider et al. publication suggest that antibodies against pS409/410-TDP-43 have therapeutic potential and further support our idea to develop fragment antigen-binding region (Fab) fragments from our antibodies to treat FTLD-TDP and ALS. We thank the reviewer for calling our attention to this work and have revised our manuscript to include the Riemenschneider et al. publication as a reference. At the same time, it is worth noting that, while both our work and that of Riemenschneider et al. use the same peptide, there is no overlap between our studies. While Riemenschneider et al. showed that antisera from a mouse injected with pS409/410-TDP-43 peptides can detect TDP-43 inclusions in FTLD-TDP patients, hybridoma were not generated to produce a monoclonal antibody against pS409/410-TDP-43. The authors did produce nine monoclonal antibodies against multiple regions of TDP-43, but none of these antibodies are specific for pS409/410-TDP-43. In contrast, our manuscript focuses on the generation and characterization of rabbit monoclonal antibodies against pS409/410-TDP-43, as well as evaluation of their utilization for investigating TDP-43 pathology both in vitro and in vivo.*

2. The major confusion raised while following the manuscript was trying to understand which species are specifically targeted by the antibodies. Although still open to debate, it could be concluded that phosphorylation of TDP-43 low complexity domain promotes insolubility (see da Silva et al. doi: 10.15252/embj.2021108443). In addition, CK1 phosphorylation may not be complete, which means that the antigens for immunization could correspond to aggregates containing mixtures of phosphorylated and nonphosphorylated TDP-43. In Figure 2, for instance, it is not clear if cell B supernatant is targeting aggregated (urea-soluble, in a broad way) rather than selectively phosphorylated TDP-43. In addition, Figure 4 suggests that the novel antibodies are targeting aggregated TDP-43. To clarify this, authors should demonstrate that aggregated, non-phosphorylated TDP-43 is not detected by the novel antibodies.

*Response: We apologize for the confusion. To generate the antibodies, we used keyhole limpet hemocyanin-conjugated pS409/410-TDP-43 peptides (CSMDSK[pS][pS]GWGM), not CK1-treated recombinant TDP-43 (rTDP-43), as the antigen to immunize the rabbit. Thus, we have ensured that all antibodies were generated from a phosphorylated antigen and not a mixture. While we included this information in the Materials and Methods section in our original submission, we have revised the manuscript to now include this information in the Results section, as well. We hope this will help clarify our process to the reader. Moreover, we appreciate the reviewer’s concern regarding whether our antibodies are truly phospho-specific or instead targeted to aggregated species. As shown in our manuscript, our antibodies consistently detect TDP-43 only under conditions where it is phosphorylated at serines 409/410 and not under conditions where these sites are absent or unmodified (Figures 1B, 2B, 3C, S2). Notably, this detection is independent of TDP-43 aggregation because our antibodies can detect both soluble pS409/410-TDP-43 peptides in the ELISA assay (Figure 3A) and aggregated TDP-43 in FTLD-TDP patients (Figures 4A, 5B, 5C).*

3. Authors generate a double Ala mutant in positions 409/410 as a control of phosphorylation of these specific sites. Instead, authors should test the possible detection by the antibodies of a truncated version of TDP-43, where both 409 and 410 (and beyond) sites are not included.

*Response: We thank the reviewer for the suggestion to make the truncated version of TDP-43 that lacks both amino acids 409 and 410 (and beyond) and agree that this would be a useful addition to our study. We generated a truncated plasmid that terminates at residue 408 (GFP-TDP-43_1-408_); given that TDP-43 contains 414 total amino acids, this truncated species lacks the last 6 amino acids. In light of the previous comment, we also decided to generate a second truncated species that harbors a mutated nuclear localization sequence (NLS). This mutated NLS causes the GFP-TDP-43_1-408-NLSmut_* *construct to accumulate in the cytoplasm, mimicking disease-associated changes in TDP-43 localization and phosphorylation. We transfected HEK293T cells with GFP-TDP-43, GFP-TDP-43_1-408,_ GFP-TDP-43_NLSmut_, or GFP-TDP-43_1-408-NLSmut_ for 48 hours and then prepared lysates for western blot. We chose our 26H10 antibody for immunoblot analysis and observed that our antibody can detect the phosphorylation of wild-type TDP-43 in cells expressing GFP-TDP-43 (Figure S2). This phosphorylation was more robust in cells expressing GFP-TDP-43_NLSmut_ (Figure S2). In contrast, our 26H10 antibody* *does not detect any signal in cells expressing GFP-TDP-43_1-408_ or GFP-TDP-43_1-408-NLSmut_ (Figure S2),* *further confirming its specificity for pS409/410-TDP-43, even in the context of TDP-43 mislocalization. We revised our manuscript to include these results in the Results section.*

Minor points:

1. Please provide an explanatory scheme of the procedure followed to produce the antibodies and the specific epitopes which are targeted.

*Response: We thank the reviewer for the suggestion. To help clarify our methods and procedure to the reader, we have included a simple explanatory schematic as Fig S1. Moreover, As discussed in #2, we used keyhole limpet hemocyanin-conjugated pS409/410-TDP-43 peptides as the antigen for generating the antibodies. As shown in Fig. 3, ELISA and western blot analyses showed that our antibodies specifically target phosphorylated TDP-43 at S409/410.*

2. Please include the sequence of the biotinylated peptides used in the dot-blots in Fig.2.

*Response: It seems our methods may not have been clear in our original manuscript and we apologize for the confusion. In the dot blot experiments shown in Fig. 2A and Fig. 3B, we used recombinant TDP-43 proteins (rTDP-43) treated with or without CK1, not biotinylated peptides. We did use biotinylated peptides in the ELISA analyses shown in Fig. 3A, and have reported the pS409/410-TDP-43 peptide sequence (which we used as an antigen) in the revised manuscript. The sequences of peptides used are shown below.*

*Non-phosphorylated S409/410-TDP-43 peptides: CSMDSKSSGWGM*

*pS409/410-TDP-43 peptides:* *CSMDSK[pS][pS]GWGM*

*pS409-TDP-43 peptides: CSMDSK[pS]GWGM*

*pS410-TDP-43 peptides: CSMDSK[pS]GWGM*

3. Please include a loading control in the western blots (Calnexin, b-actin).

*Response: We thank the reviewer for the suggestion and apologize for the oversight. For western blots using samples from cultured cells, we have included GAPDH as a loading control (Fig. 2B and Fig. 3C). Unfortunately, for western blots using urea fractions from human frontal cortex, loading controls like GAPDH or β-actin are not well detected because this fraction contains only very insoluble proteins. Nevertheless, we have updated our manuscript to now show total TDP-43 levels in the urea soluble fraction as a control. As shown in the revised Figure S3A, immunoblots showed that the levels of full-length TDP-43, which runs at ~43 kDa, are comparable between normal controls and FTLD-TDP patients. However, TDP-43 high molecular weight species and 25 kDa fragments are only observed in FTLD-TDP patients.*

4. Antibodies are used in 1:500 dilution in immunochemistry experiments. This appears too high. Please provide an explanation.

*Response: The reviewer makes a good point. We tested dilutions of 1:500, 1:1000, 1:2000 and 1:4000 and have included the results in the Results section of our revised manuscript. As shown in the revised Figure S3B, immunohistochemical analysis showed that our 26H10 antibody can detect TDP-43 pathology at all four dilutions, indicating that our 26H10 antibody has a high sensitivity.*

**Reviewer Figure 1. The rabbit bleed specifically detects pS409/410-TDP-43.** (**A‒C**) Uncropped immunoblot analysis of rTDP43 with or without CK1 treatment using the Pre-bleed (A), Bleed 1 (B) or tTDP-43 antibody (C). Arrows indicate TDP-43 oligomers and truncated forms (B, C)

5. Please show the uncropped gel in Fig. 1A, or at least the region covering molecular weights below 34 kDa. This is particularly relevant in Fig. 1B since the data with the double Ala mutant appears crucial.

*Response: We thank the reviewer for the suggestion. We revised Fig. 1A and Fig. 1B to include the region covering molecular weights below 34 kDa. We also provided fully uncropped images of the gels shown in Fig. 1A here (Reviewer Figure 1). In addition to non-phosphorylated and phosphorylated full length recombinant TDP-43 (rTDP-43) running between 43‒55 kDa (Reviewer Figure 1B, 1C), we also observed high molecular weight species (~180 kDa) and low molecular weight species (~34 kDa) in the CK1-treated samples (arrows, Reviewer Figure 1B, 1C). We believe that these bands correspond to TDP-43 oligomers and truncated forms, respectively. However, we did not see any bands below 34 kDa (Reviewer Figure 1B, 1C).*

6. In lines 219-220, reference to Fig 1B should instead be Fig 1C.

*Response: We corrected the reference to now read Fig 1C.*
